# Comparative study of psychoactive substance use pattern in romania and hungary

**DOI:** 10.1192/j.eurpsy.2021.1535

**Published:** 2021-08-13

**Authors:** C.R. Costescu, B. Nemeș, H.G. Coman, D. Cozman

**Affiliations:** Medical Psychology, “Iuliu Hatieganu” University of Medicine and Pharmacy, Cluj-Napoca, Romania

**Keywords:** Substance use disorders, drug dependece, Romania, Hungary

## Abstract

**Introduction:**

Europe’s drug situation is facing an increasing trend.

**Objectives:**

To compare the psychoactive substance use pattern in Romania and Hungary.

**Methods:**

Data was collected from the electronical databases of the 3^rd^ Psychiatry Clinic of Cluj County Emergency Hospital, Cluj-Napoca, Romania and from the Psychiatry Department of Kenezy Gyula Hospital Debrecen, Hungary. We included adult patients who had at least one hospital admission for mental and behavioral disorders due to psychoactive substance use between 01/01/2013 and 31/12/2016.

**Results:**

96 patients from Romania (80.2% males, mean age 27.8 years (18, 82)) and 816 from Hungary (71.93% males, mean age 47 years (18, 90)) were included. Romanian patients consumed more opioids (31.52% vs 4.34%, p<0.05 Chi-square Test), cannabinoids (66.3% Ro vs 13.04%, p<0.001 Chi-square Test), and synthetic drugs (declared 73.91% vs 8.21%, p<0.001 Chi-square Test), an underlying personality disorder (52.08% vs 34.06% p=0.001 Chi-square test) was more often diagnosed. Hungarian patients consumed more alcohol (89.46% vs 30.43%, p<0.001 Chi-square Test), a comorbid bipolar disorder (18.75% vs 5.2%, p=0.001 Chi-square Test), a major depressive disorder (40.8% vs 16.6% p<0.001, Chi-square Test) or an anxiety spectrum disorder (55.26% vs 7.29%, p<0.001 Chi-square test) were more often diagnosed. Overall, more than 85% had a dependence use pattern, more than 65% having multiple admissions.

**Conclusions:**

Romanian drug users are younger, prefer opioids, cannabinoids and synthetic drugs and have more often a comorbid personality disorder than Hungarian patients, who consume more alcohol and have a comorbid affective disorder.

